# Effect of minerals and heavy metals in sand samples of Ponnai river, Tamil Nadu, India

**DOI:** 10.1038/s41598-021-02717-x

**Published:** 2021-12-01

**Authors:** A. Chandrasekaran, C. K. Senthil Kumar, V. Sathish, S. Manigandan, A. Tamilarasi

**Affiliations:** Department of Physics, Sri Sivasubramaniya Nadar College of Engineering (Autonomous), Kalavakkam, Chennai, 603110 Tamil Nadu India

**Keywords:** Environmental sciences, Hydrology

## Abstract

River sand samples have been collected from Ponnai river, Tamil Nadu, India for characterization of minerals and heavy metals by different spectroscopic techniques. Initially, the samples were subjected by Fourier Transform-Infra Red (FT-IR) spectroscopic technique and infra-red absorption bands values are observed in the range of 515–520, 695–700, 775–780 cm^−1^ which shows the presence of quartz in all the samples. Similarly, infra-red peaks were absorbed for feldspar, kaolinite, calcite, gibbsite and organic carbon and confirmed by X-Ray diffraction (XRD) technique. Additionally, zircon, aragonite, magnetite and kyanite minerals were identified in the samples using only the XRD method. The concentration of heavy metals such as Pb, Cr, Zn, Ni, Hg, As, Mn, Cu has been determined by flame atomic absorption spectrometry (FAAS). An average metal concentration measured in mg kg^−1^ were: Pb 0.12, As 0.15, Hg 0.13, Cu 2.80, Zn 10.15 Cr 12.70, Ni 2.86 and Mn 104.94 and hence found in the order of Mn > Cr > Zn > Ni > Cu > As > Hg > Pb. These average values do not exceed the world average value and hence potentially do not affect the quality of sand in the river. In addition to that, presences of heavy metals are confirmed by scanning electron microscope equipped with energy dispersive X-ray spectrometry (SEM/EDS) analysis. In order to understand the possible natural and anthropogenic sources of heavy metals, multivariate statistical techniques such as Pearson correlation, principal component and cluster analysis were performed. Results obtained from the statistical techniques were good agreement with each other.

## Introduction

Sand is a naturally occurring granular material composed of finely divided rock and mineral particles and formed by the weathering of rocks. The most common constituent of sand is silica (Silicon dioxide, or SiO_2_), usually in the form of quartz. Other minerals such as feldspar, clay and carbonate minerals also dominated in the sand and sediments. FT-IR and XRD are powerful, efficient and accurate measurement techniques for mineral analysis^1,2^. In addition, many researchers have reported the mineral content of river sands throughout the world as well as in various parts of India^3–7^.

Rivers are important for water source and supports the both a way of life and livelihood for huge number of people, providing income via fishing and aquaculture. Due to industrialization and urban development, environment has been polluted through both natural and anthropogenic activities.These activities resulted in increasing quantities of contaminants into the lakes, rivers, reservoirs, and wetlands^8^. Consequently, a variety of environmental problems have cropped up and toxic metal pollution has become a major issue, especially in urban air, soils and sediments^9–11^.

River pollution is an important issue in the world which requires ongoing evaluation and revision. River pollution occurs, when unwanted materials enter into the river, changes the quality of river and anthropogenic activities such as discharge of industrial and domestic wastewater, the dumbing of sewage, washing of motor vehicles, agricultural activities, firing and mining makes the adverse effects in the river area. It also affects the aquatic animals, micro-organisms and human health^12,13^. These anthropogenic activities enhance the level of heavy metals in the river sediments and sand samples. Heavy metal (HM) pollution is inflicting rivers worldwide^14,15^, especially in developing countries^16,17^. As an important component in riverine ecosystems, sediment serves as both a sink and a source of heavy metals^18,19^. Most heavy metals quickly deposit into the sediment after entering rivers, and are much more concentrated in the sediment than in the water body of river systems^20,21^.

In recent decades, several analytical instrumental techniques can be employed for the determination of heavy metals. For example, the heavy metals Cd, Pb, Hg and Cr can be determined by Atomic absorption spectrometry (AAS), Energy-Dispersive X-ray Fluorescence (EDXRF) spectrometry, Inductively coupled plasma-Optical emission Spectrometry (ICP-OES) or Inductively Coupled Plasma-Mass-Spectrometry (ICP-MS)^22,23^. Hexavalent chromium can be determined by UV–Vis spectrometer^24,25^. On the other hand, XRF can also be used for quantification of heavy metals and total bromine^26^. In this work, heavy metals such as Pb, Cr, Zn, Ni, Hg, As, Mn, Cu is determined by Flame Atomic Absorption spectroscopic technique (FAAS) since it has high precision and rapid process in elemental analysis^27^. The detection limit for AAS is up to 0.1 μg kg^−1^ under optimum test conditions.

With its high spatial resolution, large depth field, and simple specimen preparation, scanning electron microscopes with energy dispersive X-ray spectrometry (SEM/EDS) are a suitable technique most commonly used in soil, sediment and rock characterization. The SEM/EDS mapping has been integrated to provide a new perspective of the dynamic biogeochemistry processes. Scanning electron microscopy (SEM) was used to examine the morphology of particles and aggregates in sediments of Ponnai river, Tamil Nadu. Energy-dispersive X-ray spectrometry (EDS) was also used to analyze the elemental composition and distribution, with a focus on heavy metals.

A multivariate statistical method such as the principal component analysis (PCA) and cluster analysis (CA) is a powerful tool for evaluating pollution levels among samples^28,29^. The PCA method has been widely applied in geochemical applications to identify the sources of pollution and to distinguish natural pollution from anthropogenic pollution^30,31^. When combined with PCA, CA serves as a check for results and allows for the grouping of individuals parameters and variables^32–37^.

Based on the above discussions, the main objectives of the present work is to (i) identify the primary (quartz (SiO_2_), and feldspar (Na, K)AlSi_3_O_8_) and secondary minerals (clay, carbonate and Gibbsite minerals) in the sand samples using FT-IR spectroscopic technique (ii) confirm the identified minerals by XRD technique in sand samples (iii) to determine the concentration of heavy metals such as As, Hg, Pb, Cu, Zn, Ni, Cr and Mn in sand samples using Atomic absorption spectrometry (iv) obtained results of heavy metals are compared with toxic reference values given by United State Environment Protection Agency (USEPA), average shale value (ASV) and TRV (toxicity reference value), (v) to confirm the presence of heavy metals using scanning electron microscope equipped with energy dispersive X-ray spectrometry (SEM/EDS) and (vi) to find the possible pollution sources of heavy metals whether natural or anthropogenic using multivariate statistical techniques.

## Geology of the study area

**Figure 1:**
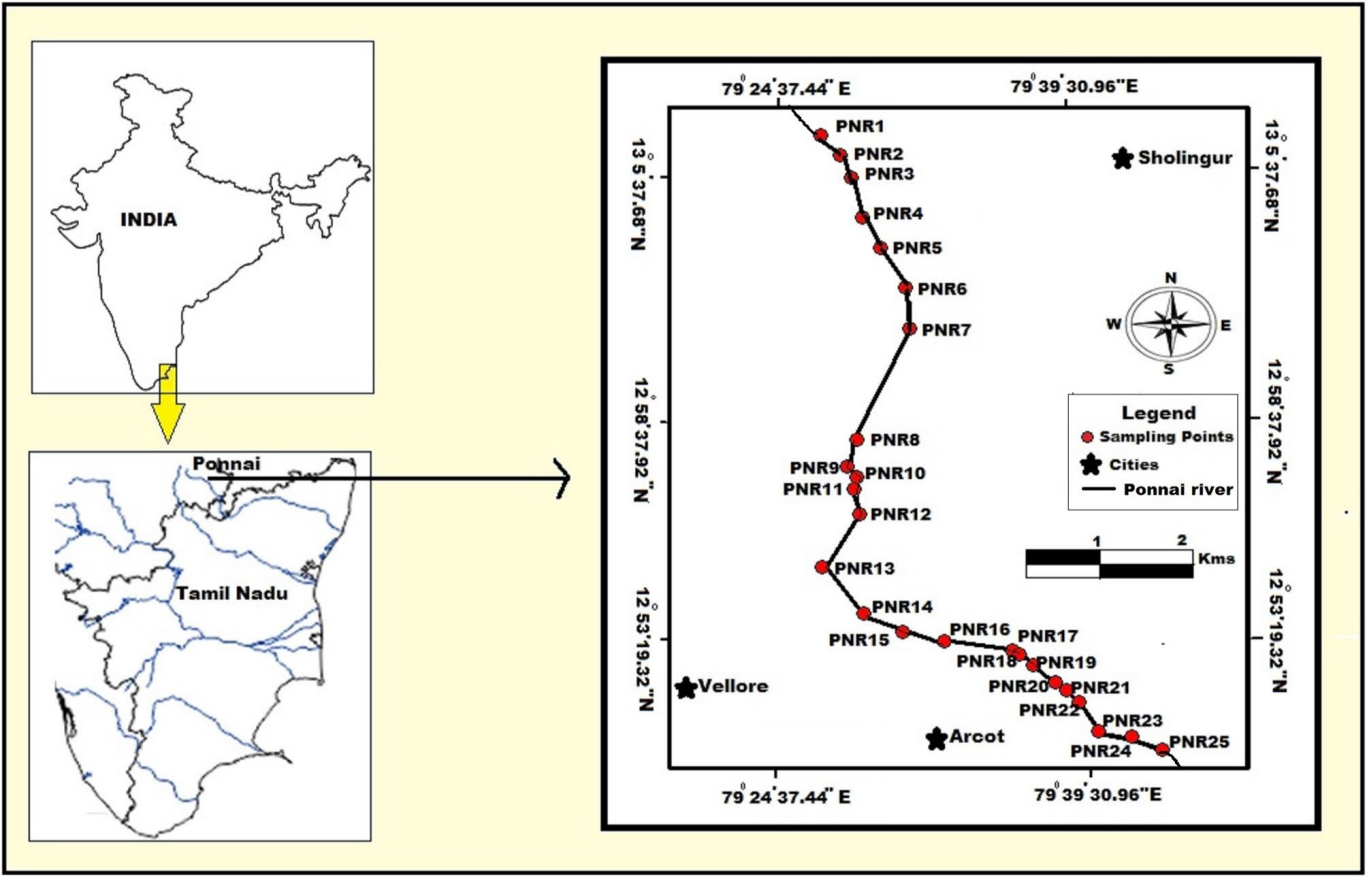
Sampling locations in Ponnai River, Tamilnadu (Map drawn using the software “MapInfo Professional version 8.5”).

Geologically, the Ponnai river flows from the North direction and ends with the East direction and sampling points are depicted in Fig. 1. It passes along the hard rock formation of Archean crystalline and metamorphic complex that overlaid by sedimentary formation. The area underwent numerous tectonic and magmatic activities during the pre-Cambrian period. Fissile hornblende biotite gneiss is the oldest rock in the study area. Charnockites are coarse grained and their color is bluish dark to grey and it occurred a few sq.km where the Ponnai river meets Palar river. It is the second largest rock type present in the area. They are massive and less weathered than the gneisses. As well as the river cross another hard rock terrain which includes Granite that’s present next to Charnockite rock. Small patches of pyroxene granulite occurred at the end of the sample location with the district border of Vellore. Geologically, the basin is underlain by rocks of Archean age consisting of granites, granite gneisses, recent alluvium and soils^38^. Sedimentary deposits are seen along the flood plain of the river that is laid on by the various hard rock formations^39,40^.

## Materials

### Sample collection

Sampling sites were selected to cover the entire stream from its source to its confluence with natural and anthropogenic activities^41^. The geographical information of each location (longitude and latitude) is noted and then stored on the internal memory of the Garmin GPS and given Table 1. The samples were collected at 25 different locations PNR1–PNR25 along the Ponnai River (Fig. 1) Tamil Nadu using a stainless steel auger which was cleaned between samples and the first subsample at each point was discarded to avoid cross contamination. In each location, five representative sub-samples, one from the center point and four from the four quadrants of the 1 m^2^ area of each point, were taken at depth 5 cm from surface of the riverbed and combined to make one composite sample representing each point on the grid. Sand samples were dried at room temperature (33 °C) and stored in clean polythene bags for further studies^42^.

**Table 1:** Geographical information of Sampling locations in Ponnai River, Tamilnadu.

S. no.	Sample ID	Geographical coordinates		Location name
		Longitude	Latitude	
1	PNR1	79° 15′ 17.964″ E	13° 7′ 17.796″ N	Ponnai
2	PNR2	79° 15′ 45.936″ E	13° 6′ 48.096″ N	Dhanalakshmai Nagar
3	PNR3	79° 16′ 1.596″ E	13° 6′ 16.452″ N	Vasur
4	PNR4	79° 16′ 17.076″ E	13° 5′ 18.06″ N	Anaicut
5	PNR5	79° 16′ 43.824″ E	13° 4′ 34.86″ N	Palleri
6	PNR6	79° 17′ 19.104″ E	13° 3′ 37.368″ N	Kondakuppam
7	PNR7	79° 17′ 24.756″ E	13° 2′ 38.148″ N	Veppalai
8	PNR8	79° 16′ 9.156″ E	12° 59′ 55.86″ N	Ekambaranellur
9	PNR9	79° 15′ 56.808″ E	12° 59′ 17.988″ N	Thiruvalam
10	PNR10	79° 16′ 8.724″ E	12° 59′ 2.76″ N	Nellikuppam
11	PNR11	79° 16′ 5.592″ E	12° 58′ 45.516″ N	Sikarajapuram
12	PNR12	79° 16′ 13.332″ E	12° 58′ 8.292″ N	Ammundi
13	PNR13	79° 15′ 21.024″ E	12° 56′ 51.612'' N	Thengal
14	PNR14	79° 16′ 19.488″ E	12° 55′ 44.472″ N	Melvisharam
15	PNR15	79° 17′ 13.812″ E	12° 55′ 17.4″ N	Navlock
16	PNR16	79° 18′ 14.4″ E	12° 55′ 4.26″ N	Veppur
17	PNR17	79° 19′ 50.304″ E	12° 54′ 51.516″ N	Arcot bridge
18	PNR18	79° 20′ 0.24″ E	12° 54′ 46.404″ N	KaspaArcot
19	PNR19	79° 20′ 19.644″ E	12° 54′ 29.52″ N	Muppathuvettiarcot
20	PNR20	79° 20′ 52.08″ E	12° 54′ 4.716″ N	Vannivedu
21	PNR21	79° 21′ 6.156″ E	12° 53′ 54.024″ N	Gudimallur
22	PNR22	79° 21′ 23.616″ E	12° 53′ 36.708″ N	Pachaimman temple
23	PNR23	79° 21′ 52.488″ E	12° 52′ 54.12″ N	Pudupadi
24	PNR24	79° 22′ 39.18″ E	12° 52′ 45.984″ N	Chennasamudram
25	PNR25	79° 23′ 22.38″ E	12° 52′ 28.164″ N	Thirumalaicherry

### Sample preparation

#### For FT-IR study

A fine homogenized powder sample of 2 mg was mixed with 40 mg of KBr in the ratio 1:20 and it’s was ground well using mortar and pestle. Before blending, required amount of KBr powder was dried at 120 °C for 6 h in an oven. If not, the broad spectral peak will appear due to unbound OH will consequentially affect the interpretation on the bound hydroxyls associated, with any of the minerals^1^. Materials required for KBr pressed-pellet method are Potassium Bromide (KBr), Acetone; die for making KBr pellets, laboratory hydraulic press for creating pressure on the confined sample, small hand agate mortar and pestle and mechanical vacuum pump. The prepared pellet was stored in a moisture free glass container before it was placed in a suitable sample holder and it was used for infrared beam for analysis.

#### For XRD study

The collected sand samples were made into fine-grained particles using agate mortar. Powdered samples are dried and sieved through 2 mm sieve. Sieved sample is homogenized in a tumbler mixer for 30 min. The prepared samples are then subjected to analysis.

#### For atomic absorption spectrometry (AAS) study

Wet digestion method was used for digestion of the river sand samples. 2.5 g of each of the air-dried, ground, and sieved samples was accurately weighted into a digestion tube. 10 ml HNO_3_ and 1 ml H_2_O_2_ were measured and added into the digestive tube and swirled gently to mix the sample properly. The digestion tubes were then placed on digestive furnace and heated at a temperature of 180 °C for 3 h. All the digests were cooled and filtered through Whatman No.42 filter paper and diluted to 50 ml by double distilled water^43^. Each sample was digested in replicates of five and transferred to acid-washed stoppered glass bottle, labeled and kept for metal analysis.

## Methods

### FT-IR analysis

**Figure 2:**
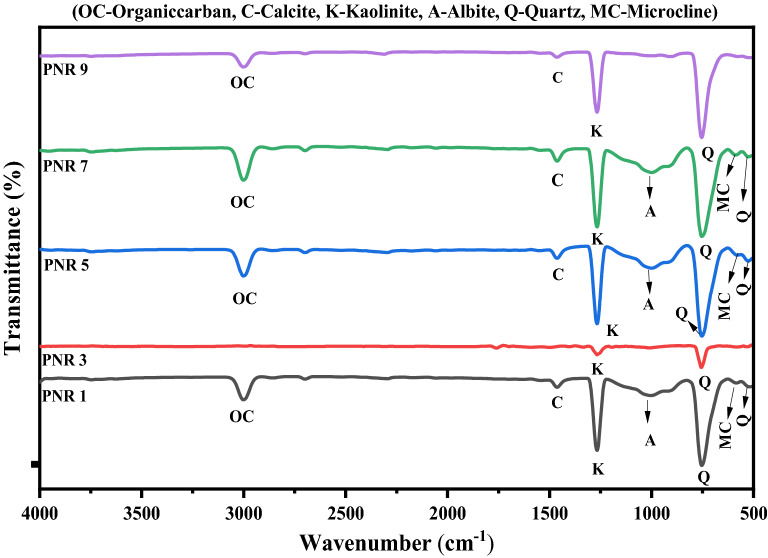
FT-IR spectrum of river sand samples PNR1–PNR9.

**Figure 3:**
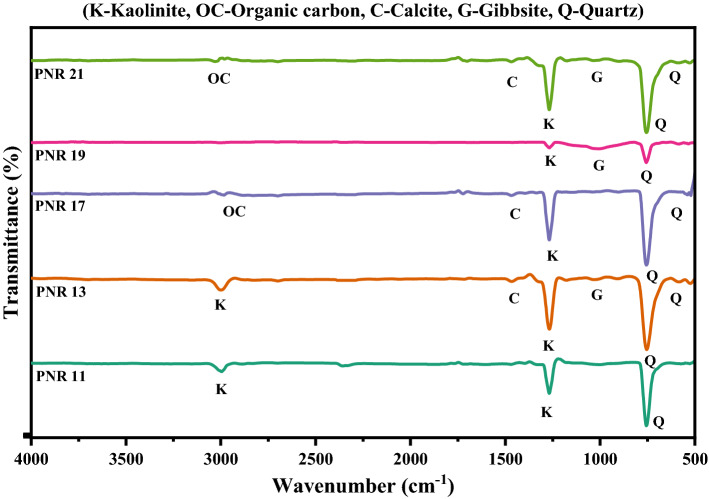
FT-IR spectrum of river sand samples PNR11–PNR21.

The Fourier transform infrared (FT-IR) spectroscopy is a powerful and well known method implemented to identify the mineral constituents present in the river sand samples. The compounds synthesized were characterized by FT-IR spectra in the region 4000–400 cm^−1^ using Perkin Elmer FT-IR spectrometer (model: Spectrum 2000). KBr was used to make a pellet format. It also has great importance in resolving the order–disorder for identifying the different functional groups in the mid-infrared area and for structural analysis present in the samples^44^. The recorded FT-IR spectrums for sand samples are given in Fig. 2 (PNR1–PNR9) and Fig. 3 (PNR11–PNR21).

### XRD analysis

**Figure 4:**
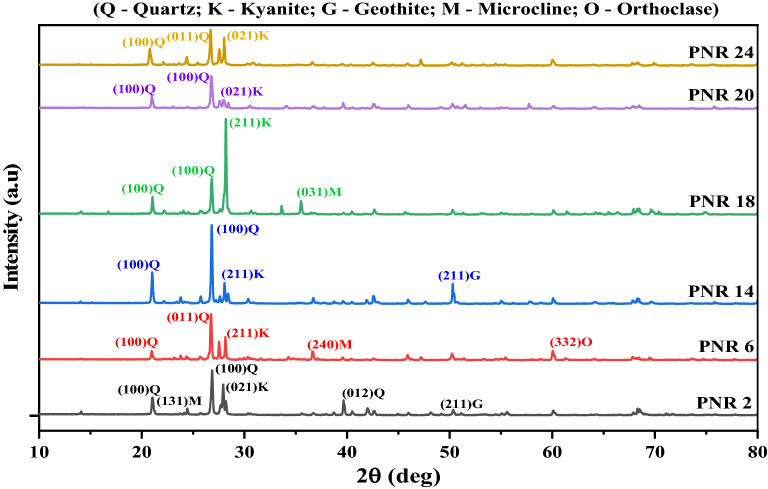
XRD spectrum of river sand samples PNR1–PNR25.

In the present study, X-ray diffraction patterns were recorded for river sand samples and given in Fig. 4. The obtained XRD patterns were compared data provided by international center diffraction data (ICDD) formerly known as joint committee on powder diffraction standards (JCPDS) for mineral identification^45,46^. In this analysis, only those peaks of the minerals, which did not overlap with sufficient intensity, were considered for identification.

### AAS analysis

In the present work, concentrations of heavy metals were determined using a Flame atomic absorption spectrometer (AAS, Analyst iCE3000, Thermo scientific, USA). Qualitative and quantitative of heavy metals are measured based on the measurement of absorption of optical radiation by atoms in the gaseous state^27^. The standard solutions for all the heavy metals under study were prepared in three to five different concentrations to obtain a calibration curve by diluting stock standard solution of concentration 1000 ppm. Regular operating conditions for sample analysis are given in Table 2. The hollow cathode lamps for Pb, As, Hg, Cu, Zn, Cr, Ni and Mn were used as radiation source and fuel was air acetylene. All the samples and standard were analyzed in multiple times and average value taken as a results.

**Table 2:** FAAS operating conditions for the determination of heavy metals in sand samples.

S. no.	Heavy metal	Wavelength (nm)	Slit width (nm)	Lamp current (mA)	Instrument mode	Flame type
1	Arsenic (As)	193.7	0.2	5	Vapour	Argon
2	Lead (Pb)	217	0.7	6	Flame	Air-acetylene
3	Chromium (Cr)	357.9	0.4	7	Flame	Nitrous oxide + Acetylene
4	Manganese (Mn)	279.5	0.2	5	Flame	Air-acetylene
5	Nicel (Ni)	232	0.7	7	Flame	Air-acetylene
6	Zinc (Zn)	213.9	0.7	5	Flame	Air-acetylene
7	Mercury (Hg)	253.7	0.7	6	Vapour	Argon
8	Copper (Cu)	324.8	0.4	5	Flame	Air-acetylene

### SEM–EDS analysis

The determination of concentration of heavy metals present in the samples was done using the Carl Zeiss Microscopy GmbH Germany (EVO 18) Energy Dispersive Xray spectrometer (SEM–EDS). This technique is being used in numerous applications for environmental science and technology. Energy dispersive X-ray spectrometry is a popular method for the determination of trace elements in geological and environmental samples. With the morphological characters obtained from SEM, supported by Energy dispersive Xray (EDS) micro analysis device, it is possible to identify elements like Na, Mg, Al, Si, Cl, K, Ca, Mn, Fe, Cr, Co, Ni, Cu, Zn, As, Se, Pb and Cd in soil and plants.

### Multivariate statistical methods

Multivariate statistical techniques could be used to identify similar origins or geochemical characteristics between the heavy metals from their inter-relationship. Hence it is performed to identify the origin of heavy metals for river sand samples using SPSS 16.0. Pearson correlation analysis was carried out to find out the relation between heavy metals. Principal component analysis (PCA) was extracted to identify the natural and anthropogenic origins to be distinguished for metals in the samples. Varimax rotation was applied to highlight the contribution of the most important variables. Then, cluster analysis (CA) was applied using average linkage method and the Euclidean distance as a measure of similarity^47^. Both techniques were applied to standardized data in order to improve interpretation and avoid misclassification**.** All of the results were generally consistent with each other.

## Results and discussion

### Mineral identification by FT-IR

The observed peak values for the minerals present in the samples are tabulated in Table 3 By using FT-IR absorption peak values; we can identify the major and minor composition in the samples, compared with the already reported literature^2,44,48–52^. The list of various minerals discussion is presented below.

**Table 3:** Observed absorption frequency in the region 400–4000 cm^−1^ of sand samples with identified minerals.

S no.	Sample ID	Silicate minerals	Feldspar		Clay mineral	Organic carbon	Carbonate minerals calcite	Gibbsite	References
		Quartz	Microcline	Albite	Kaolinite				
1	PNR1	515, 696	585	785, 990	1260, 3002	2955	1442, 2876	1003	^4,49,76–78^
2	PNR2	513, 698	589	790, 990	1260, 3000	2958	–	1005	
3	PNR3	514, 698	586	786, 990	1260, 3690	2956	1440, 2875	1005	
4	PNR4	520, 696	588	787, 995	1260, 3002	2955	1441, 2876	1000	
5	PNR5	520, 695	585	787, 995	1260, 3002	2955	1440, 2878	1003	
6	PNR6	520, 696	588	787, 995	1260, 3002	2955	1441, 2876	1000	
7	PNR7	520, 696	588	787, 995	1260, 3002	2955	1441, 2876	1000	
8	PNR8	525, 695, 775	587	784, 996	1259, 3390, 3690	2957	1445, 2878	1005	
9	PNR9	775	–	785, 990	3002	2957	1445, 2878	1000	
10	PNR10	775	–	787, 995	1260, 3005	2955	1441, 2877	1005	
11	PNR11	776	–	–	–	2956	2880	1000	
12	PNR11	775	–	787, 995	1260, 3005	2955	1441, 2877	1005	
13	PNR13	775	–	–	3002	–	–	1005	
14	PNR14	513, 698	589	790, 990	1260, 3000	2958	–	1005	
15	PNR15	520, 695, 775	587	784, 996	1259, 3390, 3690	2957	1445, 2878	1005	
16	PNR16	515, 695	–	785, 995	1260, 3000	2957	1445, 2875	1000	
17	PNR17	775	–	–	1260, 3000	2956	1440, 2875	–	
18	PNR18	775	–	787, 995	1260, 3005	2955	1441, 2877	1005	
19	PNR19	513, 698	589	790, 990	1260, 3000	2958	–	1005	
20	PNR20	775	–	787, 995	1260, 3005	2955	1441, 2877	1005	
21	PNR21	775	–	787, 995	1260, 3005	2955	1441,2877	1005	
22	PNR22	520, 696	588	787, 995	1260, 3002	2955	1441, 2876	1000	
23	PNR23	515, 695	–	785, 995	1260, 3000	2957	1445, 2875	1000	
23	PNR24	520, 696	588	787, 995	1260, 3002	2955	1441, 2876	1000	
25	PNR25	520, 696	585	787, 990	1259, 3005	2958	1445, 2876	1000	

Quartz is the most abundant and widely distributed mineral found at earth’s surface. It can be observed from the Table 2, the i.r. absorption bands values appears in the range of 515–520, 695–700, 775–780 cm^−1^ indicates the availability of quartz in the samples. Microcline (KAlSi_3_O_8_) is the triclinic low-temperature K-feldspar stable at temperatures lower than 500 °C. It is usually formed by recrystallization from feldspar, and sometimes by direct crystallization from magma and hydrothermal processes. For microline the observed peak values are 585–590 cm^−1^. The absorption peak values are 785–790, 990–995 cm^−1^ was observed for albite.

Kaolinite is a clay mineral, with chemical formula (Al_2_Si_2_O_5_ (OH)_4_). From the Table 2, the FT-IR absorption peaks for kaolinite found at 1255–1260, 3000–3005, 3385–3390, 3685–3690 cm^−1^. Absorbance at 1260 cm^−1^ is attributed to Si–O stretching of clay minerals. From the Table 2, organic carbon peaks are appeared at 2955–2960 cm^−1^. From this, a very weak absorption band found at 2955 and 2957 cm^−1^ may suggest the presence of organic carbon^53^. Calcite is a carbonate mineral which is also found in the sand samples. It can be observed from the Table 2, that i.r absorption peaks appear at 1440–1445, 2875–2880 cm^−1^ are assigned to calcite^2,53,54^. Gibbsite is an aluminum hydroxide and secondary mineral which is identified from peak in the range 1000–1005 cm^−1^. Band assignment for each frequency and minerals are also given in Table 4.

**Table 4:** Band assignments of different minerals for river sand samples.

Minerals	Frequency	Tentative assignments	References
Quartz	515	Si–O asymmetrical bending vibration	^76^
Quartz	695	Si–O symmetrical bending vibration	^4^
Quartz	775	Si–O symmetrical stretching vibration	^4^
Feldspar	585	O–Si(Al)–O bending vibrations	^4^
Feldspar	785	Si–O symmetrical stretching vibration	^77^
Feldspar	990	Si–O stretching O–H deformation	^49^
Kaolinite	1255	Si–O stretching O–H deformation	^49^
Kaolinite	3005	O–H stretching of inner hydroxyl group	^78^
Kaolinite	3390	Inner OH stretching vibrations	^4^
Kaolinite	3690	Inner surface OH stretching vibrations	^4^
Calcite	1445	Vibrations of C–O bonds in calcite	^1^
Gibbsite	1005	–	^4^
Organic carbon	2955	C–H stretching vibration	^78^

### Mineral confirmation by XRD

Recorded XRD spectrum for samples is given in Fig. 4. Using the XRD parameters, primary and secondary minerals are identified in the river sand samples. Primary mineral such as quartz (SiO_2_), and feldspar (Na, K)AlSi_3_O_8_ are identified and chemical composition of these minerals are not altered by naturally since the time of origin. Quartz is the first most abundant mineral in the all studied samples from PNR1–PNR25.

Feldspar group of minerals such as microcline feldspar, orthoclase feldspar and albite are also impartment mineral in the environment samples. In the present study, microcline and orthoclase feldspar minerals are identified in the samples PNR1–PNR5; PNR21–PNR25 and, microcline feldspar and albite are identified in the samples PNR11–PNR15. All these three feldspar group minerals that is microcline feldspar, orthoclase feldspar and albite are identified in the following samples PNR6–10–PNR15; PNR16–PNR20. Hence, the feldspar group of mineral is the second most abundant minerals in the samples.

Kaolinite is a one of the secondary mineral^55^. This mineral formation is due to the decomposition and chemical alteration of primary minerals in the samples. It is also well known clay mineral. As seen from the XRD results, kaolinite present at only few samples of PNR1–PNR15 and PNR16–20. Hence it is considered as minor distribution in the samples.

Calcite and aragonite are the carbonate minerals which are major component in the igneous rock. In this work, all the samples (PNR1–PNR25) shows the presence of calcite and aragonite found in the samples of PNR16–PNR25. Hence, the calcite is also considered as major component in the samples.

Zircon is one of heavy mineral distributed as minor constituent in earth crust and found as zirconium silicate mineral with a chemical formula ZrSiO_4_. It is also one of the primary accessories mineral and found in the samples of PNR1–PNR20. Magnetite is an Iron-oxide mineral with chemical formula Fe_3_O_4._ This mineral is identified in the river sand samples from PNR6 to PNR 25 in the study area.

Kyanite and goethite is common accessory mineral which are found in almost all the river sand samples in the study area. The mineral gibbsite was identified form FT-IR but absent in XRD because this mineral was poor in crystalline nature or not in crystalline structure^55^.

The results obtained from the XRD analysis are good agreement with FT-IR analysis for the minerals quartz, feldspar, kaolinite and calcite. Also these minerals are considered as major component in the river sand samples. Additionally, zircon, aragonite, magnetite and kyanite minerals were identified in the samples using only the XRD method.

### Concentration of heavy metals in river sands

The concentration of heavy metals in the river sand samples are reported in Table 5. As seen from Table 5, the concentration of manganese (Mn) was the highest among the heavy metals analyzed from all the sampling locations and the range obtained were found to be 78.05–168.95 mg kg^−1^ with mean of 104.94 mg kg^−1^. Chromium (Cr) is very harmful to living organisms. The hexavalent form of Cr is the most toxic. The minimum level was 5.05 mg kg^−1^ at PNR13 and the mean value (12.70 mg kg^−1^) of Cr was does not exceeded the standard values set by USEPA and toxicity reference value (TRV). The maximum concentration of Cr found in the sample at PNR25 was 31.47 mg kg^−1^ which is slightly higher than the toxicity reference value which is 26 mg kg^−1^. This high concentration of Cr can cause lethality to some aquatic species in the river system. This may be due to contamination of samples by effluents from leather industries^56^.

**Table 5:** Heavy metal concentrations in sand samples collected from Ponnai River, Tamilnadu.

Sample ID	Heavy metals (mg kg^−1^)							
	Pb	As	Hg	Cu	Zn	Cr	Ni	Mn
PNR1	0.13	0.14	0.11	1.68	9.31	11.26	1.87	80.26
PNR2	0.11	0.13	0.11	1.35	8.26	9.86	1.56	78.05
PNR3	0.11	0.13	0.14	3.21	12.6	5.69	2.1	81.29
PNR4	0.13	0.11	0.13	3.65	9.1	5.26	2.69	88.96
PNR5	BLQ	0.12	0.13	2.1	8.26	8.96	1.65	100.26
PNR6	0.12	0.14	0.11	1.8	10.26	10.63	1.89	95.89
PNR7	BLQ	BLQ	BLQ	1.44	10.86	5.42	1.9	89.91
PNR8	0.13	0.15	0.13	2.23	8.24	8.96	2.52	87.69
PNR9	0.11	0.16	0.14	3.21	9.65	6.89	3.65	128.02
PNR10	0.12	0.13	0.13	3.68	8.69	10.22	2.65	110.69
PNR11	0.11	0.13	0.11	4.21	10.12	18.56	1.89	105.56
PNR12	0.11	0.15	0.11	4.63	9.87	17.16	1.47	99.26
PNR13	BLQ	0.15	BLQ	2.21	10.26	5.05	2.43	99.56
PNR14	0.14	0.14	0.14	3.21	8.99	6.69	3.66	100.12
PNR15	0.12	0.13	0.16	1.26	13.65	12.36	4.26	108.96
PNR16	0.11	0.16	0.13	1.68	9.63	17.89	3.98	79.36
PNR17	0.13	0.18	0.16	3.67	8.46	10.99	3.56	84.56
PNR18	BLQ	0.16	BLQ	3.71	14.86	19.43	4.49	100.42
PNR19	0.11	0.13	0.13	4.25	12.6	15.69	1.59	130.26
PNR20	0.13	0.14	0.16	3.98	10.26	15.89	2.87	98.65
PNR21	0.14	0.13	0.13	1.89	11.26	16.89	3.86	152.6
PNR22	0.11	0.19	0.11	2.65	9.56	17.25	4.56	126.3
PNR23	BLQ	0.13	BLQ	1.62	8.28	12.43	2.82	78.39
PNR24	0.12	0.18	0.14	1.67	9.87	16.55	3.01	149.65
PNR25	BLQ	0.21	BLQ	4.99	10.76	31.47	4.69	168.95
Average	0.12	0.15	0.13	2.80	10.15	12.70	2.86	104.94
World average Turekian and Wedepohl (1961)	20	13	1.4	45	95	90	68	850

*BLQ* Below limit of quantification.

Cu is an essential metal for all living organisms, but is toxic at high levels. Concentration of copper measured in the sample PNR25 showed a maximum value which is the order of 4.99 mg kg^−1^. According to United State Environment Protection Agency (USEPA), maximum permissible value for Cu in the river sand is 31.6 mg kg^−1^, average shale value (ASV) is 45 mg kg^−1^ and TRV (toxicity reference value) is 16 mg kg^−1^. The observed value for Cu was found below the permissible limit set up by USEPA, ASV and TRV. The concentration of lead (Pb) ranged between 0.11 and 0.14 mg kg^−1^ with mean value of 0.12 mg kg^−1^. The obtained value of Pb in the present study was found below the permissible limit set up by USEPA, ASV, and TRV. Hence, lead (Pb) was the least abundant metal in the river samples and this reveals that few samples are free from automobile exhaust fumes and pesticides. Nickel (Ni) is a highly toxic metal even at low concentration. The concentration of Ni found in the different samples showed maximum value of 4.69 mg kg^−1^ at sample PNR25 and mean value is 2.86 mg kg^−1^. However, the concentration of Ni was found below the permissible limit at all of the sites.

The average value of Zinc (Zn) concentration in the sand samples of the River Ponnai was recorded as 10.15 mg kg^−1^. The observed value of Zn was found below the permissible limit at all locations proposed by USEPA, ASV, TRV and hence no adverse effect on aquatic biota^57^. Arsenic (As) is a highly toxic element that exists in various species and its average value found to be 0.15 mg kg^−1^ and Mercury (Hg) is a persistent environmental pollutant with bioaccumulation ability in fish, animals, and human beings and its range between 0.11 and 0.16 mg kg^−1^ with mean value of 0.13 mg kg^−1^. The concentration of As and Hg at all the locations showed that below the permissible limit given by USEPA, ASV, TRV and this reveals that there is absence of As and Hg contamination found in the study area.

The average concentration of heavy metals of Ponnai river sediments were compared with similar other works in the world and given in Table 6. The mean concentrations of all heavy metals measured in this study were significantly lower than those in the Xiangjiang River, which is one of the most polluted rivers in China^58^. The concentrations of Pb and Ni measured in this study were lower than those detected in the Gorges River in Australia^59^ and the Nile River in Egypt, all of which are heavily polluted.

**Table 6:** Comparison of metal concentration in river sediments with other countries.

S. no.	Country	River	Heavy metal concentration (mg kg^−1^)								References
			As	Pb	Cu	Zn	Hg	Mn	Ni	Cr	
1	China	Xiangjiag river	54.90	214.91	101.36	443.32	–	1805.17	57.14	120.44	^58^
2	Australia	Gorges river	11.00	67.00	30.00	157.00	–	–	13.00	39.00	^59^
3	Egypt	Nile river	–	10.36	41.64	61.70	–	774.63	48.88	72.68	^60^
4	Pakistan	Chenab river	–	18.10	8.16	33.70	–	494.00	–	–	^70^
5	Bangladesh	Turag river	–	32.78	50.40	139.48	–	–	–	43.02	^71^
6	Malaysia	Mamut river	–	23.48	583.38	61.36	–	–	170.74	–	^72^
7	France	Gardon of Ales river	27.96	61.23	–	182.60	–	–	–	–	^73^
8	Spain	Louro river	–	61.80	45.40	–	–	–	46.40	108.00	^74^
9	Singapore	Buloh river	–	12.28	7.06	51.24	–	–	–	16.61	^75^
10	India	Ponnai river	0.15	0.12	2.80	10.15	0.13	104.94	2.86	12.70	Present study

### Spatial distribution of heavy metals

**Figure 5:**
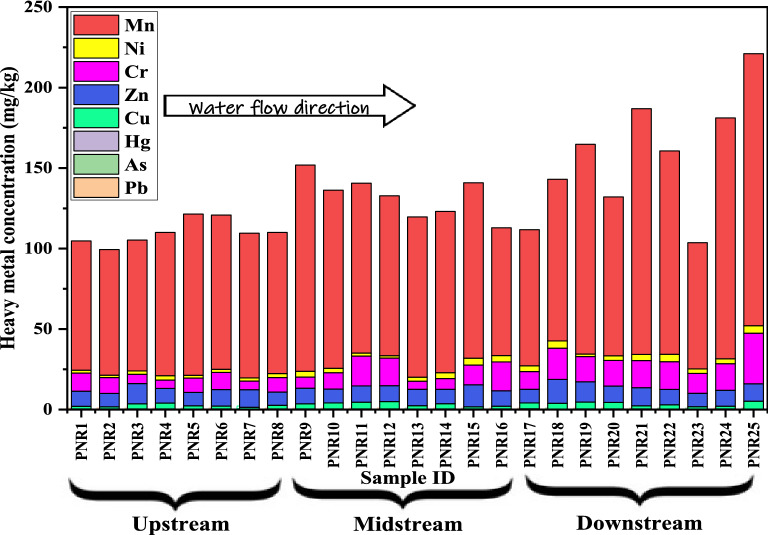
Variation of heavy metal concentration in Ponnai river, Tamilnadu.

Spatial distribution of heavy metals was studied from sampling location PNR1 to PNR25 and shown in Fig. 5. It is observed from Fig. 5, Mn has the highest concentration at PNR25 (168.95 mg kg^−1^) and then the next highest at PNR21 (150.2 mg kg^−1^). This indicates that concentration of Mn is high at downstream sampling pints (PNR17–PNR25). The total concentration of heavy metals gradually increases as the water flows PNR1 to PNR25. There is no significant difference found in the concentrations of heavy metals within the upstream (PNR1–PNR8) river area and within the midstream (PNR9–PNR16), but there is a significant difference within the downstream (PNR17–PNR25) due to discharge of sewage from households, leather industry, and transport.

### Confirmation of presence of heavy metals by SEM–EDS

**Figure 6:**
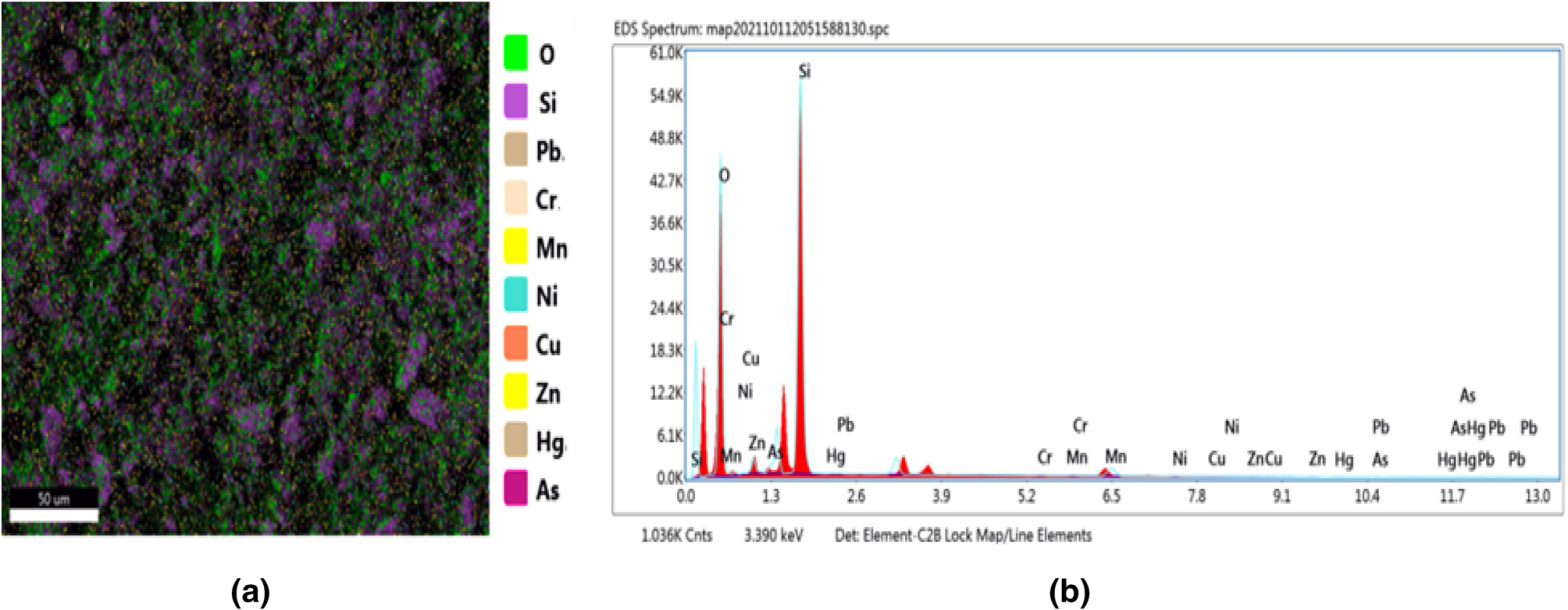
Typical SEM–EDS analysis image and spectrum for sand sample PNR25.

Studied heavy metals were found in all river sand samples mainly as small particles (< 50 µm). These particles were frequently identified inside aggregates as shown in Fig. 6a. In the view point of SEM/EDS, heavy metals (< 1 wt%) are detectable since they are concentrated in a structure^60^. SEM/EDS results confirm the presence of Zn, Cr, Pb, Ni, Cu, Mn, As and Hg in the sand samples. In addition Si and O were identified in the samples and illustrated in Fig. 6b.

### Statistical analysis of heavy metals

#### Pearson correlation analysis

Pearson correlation analysis was performed between the heavy metals to evaluate the inter-element relationship of metals and identify the source and pathways of heavy metals. The obtained results of Pearson correlation analysis for heavy metals were given in Table 6. According to Rakesh and Raju^61^, high correlation coefficient (near + 1 or − 1) means a positive correlation between two variables, and its concentration around zero means no relationship between them at a significant level of 0.05%, it can be strongly correlated, if r > 0.7, whereas r values between 0.4 and 0.7 shows moderate correlation between two different variables. Pearson correlation analysis was performed between the heavy metals and given Table 7. The obtained result reveals that there are no strong positive correlations were reordered. But a moderate correlation (r > 0.438) was found between As–Cr–Ni–Mn and Hg–Pb–Zn, indicate that the general contamination sources of these metals were primarily discharge of effluents from domestic and industry^62–64^. A very weak correlation was observed between Cu and other studied metals at p < 0.01. This indicates that Cu was derived in part from natural source (local soil or rock)^63^. Zn showed poor correlation with all other metals except Hg (0.438), which may be due to the influence of transport activities near to the river area^65,66^.

**Table 7:** Pearson correlation analysis among the heavy metals.

Correlations								
Variables	As	Hg	Cu	Zn	Cr	Ni	Mn	Pb
As	1							
Hg	0.153	1						
Cu	0.323	0.019	1					
Zn	− 0.055	**0.438**	0.150	1				
Cr	**0.547**	− 0.201	**0.400**	0.271	1			
Ni	**0.511**	− 0.079	0.077	0.277	**0.440**	1		
Mn	**0.490**	− 0.031	0.287	0.258	**0.583**	**0.433**	1	
Pb	0.015	**0.464**	− 0.065	− 0.207	− 0.189	0.156	0.008	1

Significant values are in bold.

#### Principal component analysis

Principal component analysis (PCA) identifies the potential sources of heavy metal contamination. As part of this procedure, correlation matrixes are prepared between heavy metal variables, PCs are extracted and possible rotation is performed to reach a final solution with simpler PCs. In principal component analysis, the PC1 tends to be more general, representing the most important common part of the variables analyzed. This PC1 is the best summary of the linear relationship exhibited by the data. The PC2 is independent of the first one (orthogonal), considering only the residual variance not included in the PC1, and so successively for the other axes. In this study it was decided to retain two factors for interpretation, accounting for approximately 36.59% of the total variance. To obtain more reliable information about the relationships between the heavy metals, principal component analysis was performed using varimax rotation method using Kaiser Normalization and extracted data loadings are given in Table 8. The results of PCA indicated that all the heavy metals are well represented by two components^64^. The principal components with eigenvalues greater than 1 were considered to be relevant^32^, which explains approximately 36.59% of the total variance for the data. Components with factor loadings above 0.75, between 0.5 and 0.75, and between 0.3 and 0.5 were considered to be strong, moderate and weak, respectively^33^. As shown in Table 8, PC1 included As, Cr, Ni, Mn; PC2 included Hg, Zn, Pb. This implies that heavy metals As, Cr, Ni, Mn, were derived from anthropogenic activities such as sewage from, domestic, leather industry and transport^63,67^.

**Table 8:** Rotated principal component analysis for heavy metals.

Variables	PC1	PC2
As	**0.435**	0.126
Hg	− 0.058	**0.462**
Cu	0.321	0.262
Zn	0.277	**0.532**
Cr	**0.736**	0.362
Ni	**0.459**	0.185
Mn	**0.993**	− 0.121
Pb	− 0.021	**0.513**
% of variance explained	**25.34**	**11.25**

Significant values are in bold.

#### Cluster analysis

**Figure 7:**
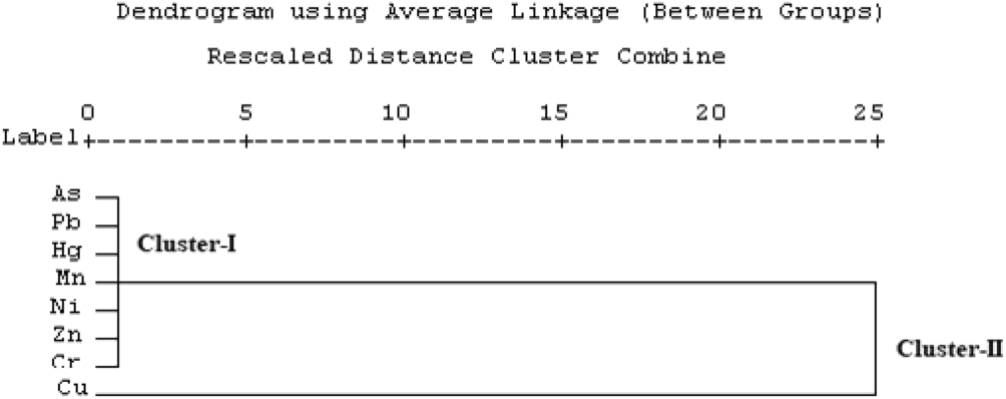
Clustering of heavy metals in river sand samples.

According to Kannel et al.^68^, the cluster analysis is a multivariate statistical technique and commonly used in several environmental studies to identify groups or clusters of same variables based on similarities. CA, which involves the evaluation of proximity matrix of squared Euclidean distance along with an agglomeration schedule for clustering similar variables. This method is regarded as very efficient and yields clearly structured and relatively stable clusters^69^. In the present work, the cluster analysis was performed using the heavy metal data set and presented in a two-dimensional dendrogram plot as shown in Fig. 7.

This dendrogram contains two clusters. Cluster I consists As, Pb, Hg, Ni, Zn and Cr. This first cluster group of metals (As, Pb, Cr, Ni, Hg, and Zn) shows the high similarities which imply that these heavy metals were mainly derived anthropogenic activities such as industrials and transport activities. Cluster II consist only Cu which indicate that Cu is derived from weathering of parent rock in the study area. These results are good agreement with PCA and Pearson correlation analysis.

## Conclusion

Ponnai river sand samples were characterized by different spectroscopic techniques to determine the presence of minerals and heavy metals. From the results of FT-IR, it is concluded that the minerals like quartz, feldspar, gibbsite and calcite are major constituent and kaolinite is the minor constituent in the samples. The presence of these minerals has also confirmed by XRD technique. This XRD treatment of samples shows that presence of accessory minerals such as zircon, aragonite, magnetite and kyanite in the samples. The identified peaks of FT-IR and XRD for minerals indicate that sample quality is not affected by anthropogenic activities. Further, among the determined heavy metals manganese (Mn) was the most abundant and lead (Pb) was the least abundant heavy metal found in study area. The total concentration of heavy metals gradually increases as the water flows from PNR1 to PNR25. The maximum concentration of Cr found in the sample at PNR25 is 31.47 mg kg^−1^ which is slightly higher than the toxicity reference value which is 26 mg kg^−1^. This may be due to contamination of samples by effluents from leather industries located near to the study area. Using SEM/EDS, heavy metals (< 1 wt%) are detected SEM/EDS results confirms the presence of Zn, Cr, Pb, Ni, Cu, Mn, As and Hg in the sand samples. The result of multivariate statistical methods reveals that studied metals As, Cr, Ni, Mn, Hg, Pb were deposited in samples due to discharge of effluents from domestic and industry while Zn was derived from transport activities. In addition to that, Cu was derived in samples due to weathering of local soil or rocks in the study area. Hence, the results of this study indicate that that quality of the sand samples is altered by both natural and anthropogenic activities daily and needs continuous monitoring to establish the pollution level of the study area.
